# Supplementary material for: Vitamin K-dependent carboxylation in osteoblasts regulates bone resorption through GAS6 in male mice
Source: Bone Res. 2026 Apr 28;14:48. doi: 10.1038/s41413-026-00528-2 (PMC13121459; doi:10.1038/s41413-026-00528-2)
Supplement: Supplementary file 1 — Supplementary figures and tables [file 41413_2026_528_MOESM1_ESM.pdf]

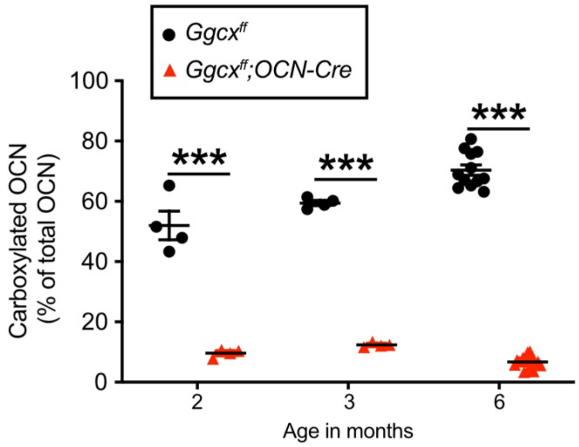

Figure S1. *Ggcx* inactivation in osteoblasts markedly reduced osteocalcin carboxylation level.

Serum level of carboxylated osteocalcin (OCN) expressed as a percentage of total osteocalcin in *Ggcx*^{ff} and *Ggcx*^{ff}*;OCN-Cre* male mice at the indicated ages (n=4-17). ***p < 0.001.

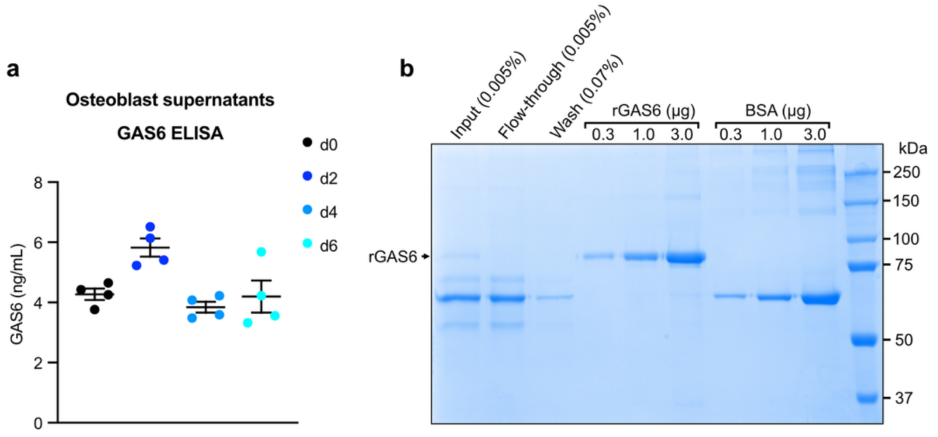

Figure S2. Analysis of GAS6 in osteoblast culture and purification of recombinant carboxylated GAS6. (a) GAS6 concentration was quantified using a specific ELISA in the supernatant of osteoblasts cultured in pro-osteoclastogenic conditions (i.e., VitD$_3$ and PGE$_2$) for the indicated times (d0-d6: day 0 to 6; n=4). **(b)** Coomassie stained SDS-PAGE gel representing the different steps of the purification. Recombinant 6×HIS tagged GAS6 (rGAS6) was produced from HEK293 cells in the presence of vitamin K$_1$ to ensure maximal carboxylation. The cell supernatant (Input) was collected and GAS6 purified by nickel affinity chromatography. Following binding, GAS6 was depleted from the media (Flow-through). After extensive washes (Wash), rGAS6 was eluted using an imidazole-containing buffer, and the purified protein was dialyzed against PBS. The concentration of the purified rGAS6 was next determined using a BSA standard (BSA).

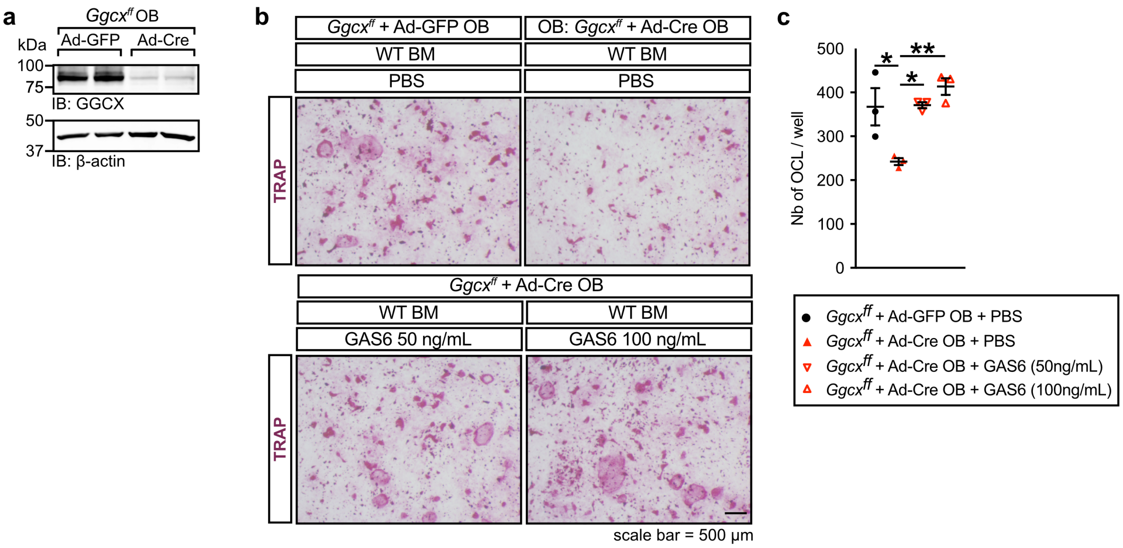

Figure S3. Carboxylated GAS6 rescues ex vivo osteoclastogenesis in *Ggcx* inactivated osteoblast co-cultures. (a) Western blot analysis of extracts from *Ggcx^ff* osteoblasts transduced with Ad-GFP or Ad-Cre. **(b)** Representative TRAP staining of *Ggcx^ff* osteoblasts (OB) transduced with Ad-GFP (control) or Ad-Cre (knockout), and co-cultured with WT bone marrow cells (BM) for 7 days in the presence of prostaglandin E_2 (PGE_2; 10^{-6} M) and 1,25 vitamin D_3 ($VitD_3$; 10^{-8} M), with or without recombinant carboxylated GAS6 at 50 ng/mL or 100 ng/mL. **(c)** Quantification of the number of TRAP+ osteoclasts per well (Nb of OCL/well) (n=3). One-way ANOVA with Bonferroni's posttests was used in (c). **$p < 0.01$, *$p < 0.05$.

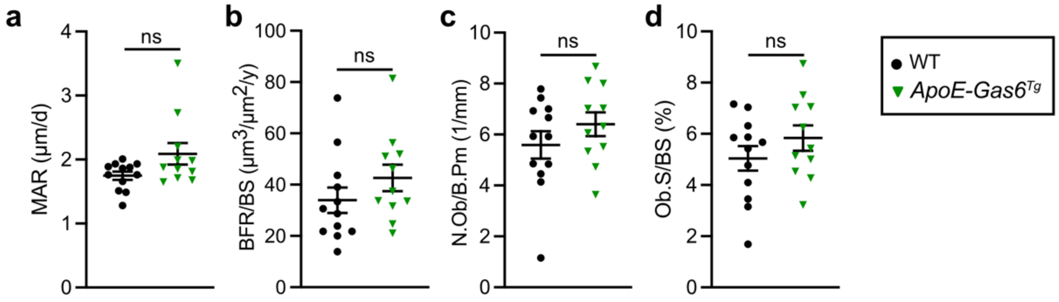

Figure S4. Increased circulating GAS6 level does not impact bone formation parameters. Bone histomorphometry analysis of lumbar vertebrae in six-month-old *WT* (non-transgenic littermates) and *ApoE-Gas6^{Tg}* male mice (n=11-12). **(a)** Mineral apposition rate (MAR). **(b)** Bone formation rate over bone surface (BFR/BS). **(c)** Number of osteoblasts per bone perimeter (N.Ob/B.Pm). **(d)** Osteoblast surface over bone surface (Ob.S/BS). ns: non-significant.

Table S1. List of oligonucleotides used in this study.

Gene	Forward Sequence 5'-3'	Reverse Sequence 5'-3'	Purpose
Ggcx	TTGACCCTCGTGTGGACATC	AATCTGCAATGAAGACCACC	qPCR
Vkorc1	ATTACCGCGCGCTCTGCGA	AAGAACAGGATCCAGGCCAG	qPCR
Hprt	TCAGTCAACGGGGGACATAAA	GGGGCTGTACTGCTTAACCAG	qPCR
Rankl	AAGATGCGACGTACTTTGGG	CGTGGGCCATGTCTCTTAGT	qPCR
Opg	GAAAGACCTGCAAATCGAGC	TTGTGAAGCTGTGCAGGAAC	qPCR
Csf1	GCAAGGGACTCACTAGCCTG	TATGCCTTTACGGGAAGTCG	qPCR
Bglap	CAGACAAGTCCCACACAGCA	CTTGGCATCTGTGAGGTCAG	qPCR
Mgp	GGCGAGCTAAAGCCCAAAAG	GTAGTCATCGCAGGCCTCTC	qPCR
Ucma	CAAGCGGTCTCCTAAGTCCC	TGGCGGTTGTAGAGGTAGGA	qPCR
Postn	AAGGCGAAACGGTGACAGAA	TCGTTCTTCCCGAGTCTGTT	qPCR
F2	TGGAAGGTCGCTGTGCTATG	CAGAGCGAGGAGTCATCACC	qPCR
F7	GAGGACTACACGCTACAGCC	CGGTCACTATCCATCTGGCG	qPCR
F9	GCAAAACCGGGTCAAATCCC	AGACAGTGGGCAGCAGTTAC	qPCR
F10	CACTGCCGTCCTTGACCAC	TTGGCACGTTCCCGGTTAAT	qPCR
ProC	GCGCTACCTGGACGAAATTG	GAACACTGGGTCAGGATGGG	qPCR
ProS1	GTGAGGGTATCCCAGTGTGC	CATCACGAAGCGCAATCAGG	qPCR
ProZ	CCCTGACTTCCGAACACATCA	CGACTCCTCGTCATAACGCAT	qPCR
Gas6	ATGAAGATCGCGGTAGCTGG	CCAACTCCTCATGCACCCAT	qPCR
Prrg1	CCAGTCACTTCCTCTGTTGGTTT	ACTAAAACGCTACCCAAGAGCC	qPCR
Prrg2	AGGCGTTTTCCTCGTGCTAA	AGGATCCCAGAGGTCAGTCC	qPCR
Prrg3	GCTCTGTGAGGGGTCTCGAA	AAGAAGCATCATGGCTGTATTTCT A	qPCR
Prrg4	CCGCCTCCTGAACAATAGGT	ATGGCCGCCTTTTACACTTG	qPCR
Actb	GACCTCTATGCCAACACAGT	AGTACTTGCGCTCAGGAGGA	qPCR
MerTK	GGTTCTGGCCCCACTGCTAC	CAGAGAATGGCCTGTGGTTGA	qPCR
Axl	CCAGTCACAGGACACAGCTC	ATACCCACCCCATCGTCTGA	qPCR
Tyro3	ACGATCTCCAGCTACAACGC	TTGTCTGAAAGGGCACCCAG	qPCR
Acp5	AGTCCTGCTTGTCCGCTAAC	CCTAAAAGGGGTGAGCCTGG	qPCR
Clcn7	TCTCGCTTGAGTGATGTTGACC	GACTGGCTGTGGGAAAGGAA	qPCR
Ctsk	CGTGCAGCAGAACGGAGGCA	GTCCTACCCGCGCCACTGCT	qPCR
Dc-stamp	TTGCCGCTGTGGACTATCTG	GAATGCAGCTCGGTTCAAAC	qPCR
Ggcx $^{flox/flox}$	TCATTGAGTCCTTCCCGAAC	TCCAAGTGCGTCTTTAACTCC	Genotyping
Oc-Cre	CAAATAGCCCTGGCAGATTC	ACGCCTGGCGATCCCTGAACAT	Genotyping
Bglap +/-	TGGAGTGGTCTCTATGACCT	TTCCTTGACCCTGGAAGGTG	Genotyping
	TTGTGCTGGGGTGGTTTCTG	AGCCTTCCCCAACCCCTATT	
Ctsk-Cre	GCGGTCTGGCAGTAAAAACTAT C	GTGAAACAGCATTGCTGTCACTT	Genotyping
tdTomato $^{flox/flox}$	AAGGGAGCTGCAGTGGAGTA	CCGAAAATCTGTGGGAAGTC	Genotyping
	GGCATTAAAGCAGCGTATCC	CTGTTCCTGTACGGCATGG	
ApoE-Gas6	AAGGCTAACCTGGGGTGAGG	AAGTTCTGAACACATTTGGCGA	Genotyping

Table S2. List of antibodies used in this study.

Antibody	Source	Catalog #	Application	Dilution
Rabbit anti-GGCX	ProteinTech	16209–1-AP	WB	1/1000
Rabbit anti-VKORC1	Ferron et al. 2015 (Ref 31)	Custom made	WB	1/1000
Mouse anti-β-Actin	MilliporeSigma	A1978	WB	1/2000
Rabbit anti-phospho-AXL (Y702)	Schott et al. 2024 (Ref 38)	Custom made	WB	1/1000
Goat anti-Axl Antibody (C-20)	SantaCruz	sc-1096	WB	1/500
Rabbit anti-phospho-AKT (Ser473)	Cell Signaling	9271	WB	1/1000
Rabbit anti-AKT (pan) (C67E7)	Cell Signaling	4691	WB	1/1000
Mouse anti-MERTK	Cell Signaling	38102	WB	1/1000
